# Suprascapular Neuropathy around the Shoulder: A Current Concept Review

**DOI:** 10.3390/jcm9082331

**Published:** 2020-07-22

**Authors:** Federico Bozzi, Sergi Alabau-Rodriguez, Sergi Barrera-Ochoa, Atesch Ateschrang, Anna J. Schreiner, Juan Carlos Monllau, Simone Perelli

**Affiliations:** 1 Department of Orthopaedics and Traumatology, Fondazione Poliambulanza (Brescia)—Università Cattolica del Sacro Cuore, 00168 Rome, Italy; 2 Institut Català de Traumatologia i Medicina de l’Esport (ICATME)—Hospital Universitari Quiròn-Dexeus. Universitat Autònoma de Barcelona, 08028 Barcelona, Spain; sralabau89@gmail.com (S.A.-R.); dr.barreraochoa@gmail.com (S.B.-O.); JMonllau@parcdesalutmar.cat (J.C.M.); perelli.simone@gmail.com (S.P.); 3 Orthopedic department, Gemeinschaftsklinikum Mittelrhein, 56073 Koblenz, Germany; ateschrang@gmx.de; 4 Department of Traumatology and Reconstructive Surgery, BG Trauma Center Tübingen, Eberhard-Karls University of Tübingen, 72076 Tübingen, Germany; annajschreiner@yahoo.de; 5 Department of Orthopaedic Surgery, Hospital del Mar. Universitat Autònoma de Barcelona (UAB), 08028 Barcelona, Spain

**Keywords:** suprascapular nerve palsy, spinoglenoid notch, suprascapular notch, Arthroscopic nerve release, shoulder neuropathy, over-head activities

## Abstract

Suprascapular neuropathy is an uncommon but increasingly recognized cause of shoulder pain and dysfunction due to nerve entrapment. The aim of this review is to summarize some important aspects of this shoulder pathology. An extensive research was performed on PubMed and Clinical Key. The goal was to collect all the anatomical, biomechanical and clinical studies to conduct an extensive overview of the issue. Attention was focused on researching the state of art of the diagnosis and treatment. A total of 59 studies were found suitable and included. This condition is more frequently diagnosed in over-head athletes or patients with massive rotator cuff tears. Diagnosis may be complex, whereas its treatment is safe, and it has a great success rate. Prompt diagnosis is crucial as chronic conditions have worse outcomes compared to acute lesions. Proper instrumental evaluation and imaging are essential. Dynamic compression must initially be treated non-operatively. If there is no improvement, surgical release should be considered. On the other hand, soft tissue lesions may first be treated non-operatively. However, surgical treatment by arthroscopic means is advisable when possible as it represents the gold standard therapy. Other concomitant shoulder lesions must be recognized and treated accordingly.

## 1. Introduction

Suprascapular neuropathy is of increasing interest as a cause of shoulder pain and weakness. It was first described by the Frenchman Andre Thomas in 1936, and further defined by Kopell and Thompson in 1959 [1,2]. It is considered a rare cause of shoulder pain even though the real incidence rate remains unknown and so may be underestimated [3,4]. The difficulty is to remember this specific and other coexisting shoulder pathologies [5]. However, the diagnosis and recognition rates of this disease are increasing and might represent 1%–2% of all causes of shoulder pain [6]. It mainly occurs in patients under 40 years of age and prevalence seems to extend widely from 12% to 33% in all athletes, representing an important cause of shoulder pain in overhead athletes [6,7]. Several studies found infraspinatus muscle impairment, suprascapular muscle atrophy and weakness in 20% to 45% of volleyball athletes [4,5,8]. Another one third of all those volleyball athletes were asymptomatic [9,10]. Some reports investigate the correlation between suprascapular neuropathy and retracted rotator cuff tears. In any case, it is still impossible to define how suprascapular neuropathy contributes to symptoms in patients affected by massive cuff-tear lesions. Moreover, some authors consider this condition as a possible cause of shoulder pain and weakness in patients with symptoms suggesting a rotator cuff tear while having normal imaging [3,11,12,13]. Only anecdotal reports of iatrogenic cases after supraclavicular block for upper extremity surgery have been described [14,15].

## 2. Materials and Method

An extensive research was performed on PubMed and Clinical Key. Search terms included suprascapular nerve palsy, spinoglenoid notch, suprascapular notch, arthroscopic nerve release, shoulder neuropathy and overhead activities. Additionally, citation tracking was performed by manually screening the reference lists of eligible studies. Reviewers assessed the studies considering whether they met the following inclusion criteria: anatomical study paper, clinical trial, systematic literature review and meta-analysis. The only exclusion criterion was: article not written in English. The goal was to collect all the anatomical, biomechanical and clinical studies in order to have an extensive overview of the issue. Attention was focused on researching the state of art of the diagnosis and treatment. A total of 66 studies were found but only 59 of them were found suitable and included.

### 2.1. Anatomy

**Figure 1:**
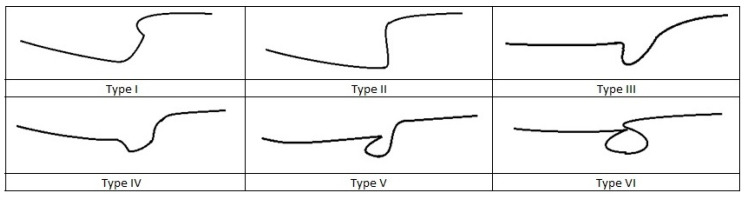
Rengachary morphothypes classification of the suprascapular notch.

The suprascapular nerve (SN), a mixed nerve that includes sensory and motor fibers, arises from upper trunk of the brachial plexus (C5, C6 and sporadically C4 roots). It runs laterally through the posterior cervical triangle and backwards to the clavicle, reaching the superior border of the scapula at the suprascapular notch underneath the superior transverse scapular ligament. The suprascapular artery and vein pass over the transverse ligament [5]. The suprascapular notch represents the most common site of compression of the suprascapular nerve. Several anatomic variations of the notch have been described that range from a simple bony tunnel to a wide and soft bony depression. It has been classified into 6 morphotypes by Rengachary (Figure 1). In type IV, the transverse scapular ligament is ossified and may form a nearly complete or complete bony foramen. This morphotype can compress the suprascapular nerve and increase the risk of entrapment [2,16,17]. After passing through the suprascapular notch, the nerve passes laterally along the supraspinous fossa, emitting motor branches to the supraspinatus muscle and receiving sensory branches from the coracohumeral and coracoacromial ligaments, the subacromial bursa and the acromioclavicular and glenohumeral joints [18,19,20]. It then proceeds underneath the supraspinatus muscle and reaches and passes through the spinoglenoid notch under the spinoglenoid ligament (or inferior transverse scapular ligament). The spinoglenoid notch, a bony depression at the lateral third of the scapular spine, is considered the second typical compression site of the suprascapular nerve [10]. The anterior coraco-scapular ligament (ACSL) is an independent ligament extending along the anterior side of the suprascapular notch below the superior transverse scapular ligament (STSL). Avery et al. recently described different anatomic variations while pointing out that the presence and the position of this specific ligament as well as the location of the suprascapular bundle vessels can cause compression or protect the suprascapular nerve from compression [21]. The spinoglenoid ligament is present in 14% to 100% of patients and links the spine of the scapula, the glenoid neck and the posterior shoulder capsule. This capsular attachment causes ligament tightening during cross-body adduction and internal rotation of the limb and may cause dynamic nerve compression [5,22]. From this point, the nerve proceeds downwards and spreads innervation to the infraspinatus muscle. Suprascapular nerve sensitive branches arise proximal to the spinoglenoid notch and innervate the posterior and superior shoulder regions and contribute to glenohumeral joint proprioception. Additionally, secondary sensitive branches innervate the coraco-humeral and coraco-acromial ligaments, the subacromial bursa and the acromioclavicular joint [10,23,24,25].

### 2.2. Aetiology

Suprascapular nerve entrapment can occur due to different pathologies at the suprascapular notch and/or spinoglenoid notch [2]. Compression is defined as primary when it is caused by dynamic entrapment of the nerve whereas it is called secondary when it is provoked by space occupying lesions, traumatic conditions, post traumatic disorders and systemic conditions. Other possible causes of suprascapular neuropathy are hormonal alterations or iatrogenic conditions [17,26]. A complete summary of the causes is provided in Table 1.

**Primary**: This etiology is relatively common in overhead athletes, such as in basketball, volleyball and baseball. The reported prevalence of suprascapular neuropathy in elite volleyball players is up to 33% in the dominant arm [27]. Repeated throwing results in a backward and forward rotation of the scapula. This significant mechanical strain can cause suprascapular nerve compression [9]. The specific mechanisms are yet not fully understood, but some hypotheses have been put forward. The suprascapular nerve gets stretched between two relatively fixed sites, the brachial plexus medially and the infraspinatus muscle laterally. Nerve compression by the suprascapular ligament is enhanced by depression, retraction and overhead abduction of the shoulder or extreme scapular motion, as in the case of repetitive pitching or throwing. Compression at the spinoglenoid notch may be caused by compression of the motor branch of the suprascapular nerve by the supraspinatus or superior side of the infraspinatus muscle adjacent to the scapular spine when the shoulder is extremely abducted or internally and externally rotated [3,10,11,18,28,29]. All these motions occur frequently during throwing or pitching. Accordingly, there is an additional association between increased shoulder range of motion and isolated infraspinatus muscle weakness [30]. Recent studies proposed another mechanism related to a tightening of the spinoglenoid ligament that cause compression of the nerve at the end of an overhead serve [4,10,22,31,32]. Rengachary et al. proposed the hypothesis of the so called “sling effect”. It consists of repeated kinking of the nerve against the borders of osteofibrous tunnel (suprascapular notch) during shoulder motion, causing suprascapular neuropathy [1,8,21,23]. Some authors have advanced two hypotheses of vascular etiologies in athletes. One speculates on intimal lesions of the suprascapular or axillar artery resulting in microemboli in the vasa nervorum of suprascapular nerve [3,5,8,33]. The second contemplates a dilatation of the suprascapular veins that could lead to nerve compression at the spinoglenoid notch [28].

**Secondary**: Space occupying lesions, such as cysts or tumors, can compress nerves at either the suprascapular notch or spinoglenoid notch [4,10,34]. Paralabral cysts are related to labral tears and glenohumeral instability. Other causes that may lead to compression are the above-mentioned suprascapular notch and anterior anatomical variations of coraco-scapular ligament, degenerative conditions like suprascapular ligament ossifications or traumatic conditions like suprascapular notch fractures, scar tissue after a distal clavicle fracture and shoulder dislocations or distraction. Post-traumatic conditions like arthritis, heterotopic ossifications and iatrogenic anesthesiological injuries or shoulder injections have also been described [1,3,14,15,23,35,36]. Furthermore, surgical procedures, such as distal clavicle excision or any posterior approach to the shoulder, can injure the nerve [37,38]. Recent research suggests rotator cuff tears as a cause of suprascapular neuropathy. Some authors have shown that medial retraction of the supraspinatus tendon causes a reduction in the angle along the nerve course with increased traction of the nerve at the spinoglenoid notch [39]. There is an additional group of secondary etiologies provoked by systemic conditions. It includes a wide range of conditions including diabetes, pregnancy, hypothyroidism and viral neuritis. Although these conditions are quite frequent, they very rarely affect the suprascapular nerve.

### 2.3. Clinical Evaluation

**Figure 2:**
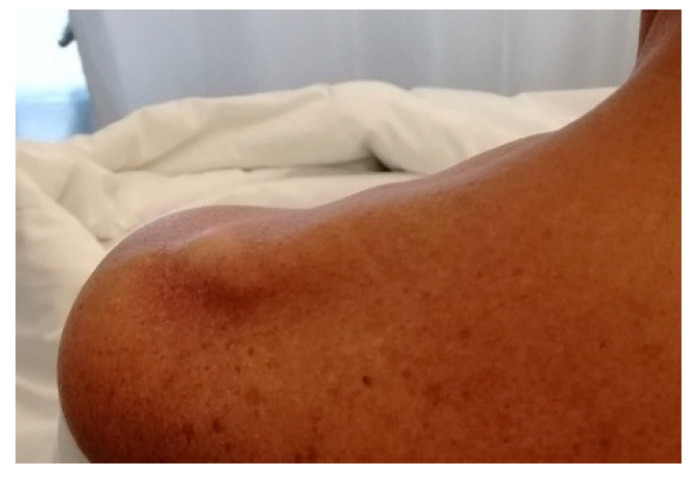
A clinical photograph of a patient with suprascapular nerve compression shows the resultant atrophy in the supraspinatus fossa.

**Figure 3:**
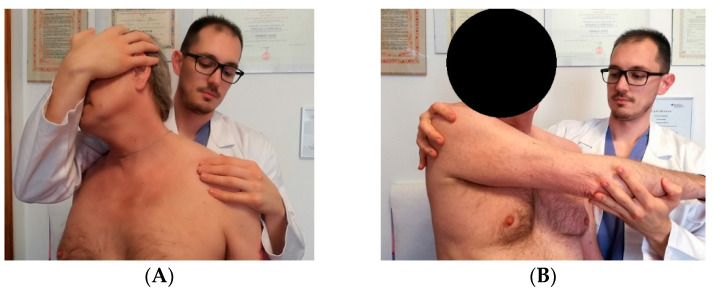
(**A**) Suprascapular stretch test; (**B**) Cross-arm adduction test.

Diagnosis of suprascapular nerve entrapment is difficult and must be considered in all patients with unexplained shoulder pain, especially in patients involved in repeated overhead activities [23]. The medical history and clinical evaluation are almost never pathognomonic because clinical presentation of the suprascapular neuropathy often mimics other more common shoulder pathologies, radiculopathy and a plexus pathology, with which the suprascapular neuropathy can coexist. This is the reason for missed, misdiagnosed or late clinical confirmation [10]. Few years ago, suprascapular entrapment was considered a diagnosis for exclusion by some authors [4,11]. After a focused anamnesis emphasizing a history of penetrating trauma, previous shoulder surgeries, anesthetic shoulder injections or overhead activities, a clinical examination should be made. Symptoms can start suddenly after a specific shoulder trauma or insidiously without any history of injury. In these cases, the clinician should consider the differential diagnosis along with neuralgic amyotrophy and the hourglass-like constriction neuropathy. Neuralgic amyotrophy is a rare neurological disorder that affects the peripheral nervous system. It is characterized by an acute and sudden onset of pain in the upper limb followed by multiple atrophy and motor weakness and slow recovery. On the other hand, hourglass-like constriction neuropathy is considered a neurological disorder brought on by fascicular constriction of one or more peripheral nerves, unrelated to intrinsic or extrinsic compression or trauma. The presentation often mimics suprascapular nerve palsy. The diagnosis is challenging as it is not observed in conventional magnetic resonance imaging (MRI) and is usually detected during exploratory surgery, which has even been proven to be beneficial. Patients with a suprascapular nerve palsy typically report a non-specific, deep, dull and continuous pain located in the upper-posterior-lateral region of the shoulder. In some instances, there is accompanying neck and arm irradiation, with or without suprascapular muscle atrophy (Figure 2) and weakness. A complete glenohumeral and acromioclavicular joint assessment is called for to exclude other concomitant pathologies [10,40]. Moreover, both shoulders must be inspected comparatively. Patients with spinoglenoid notch compression often present isolated infraspinatus muscle atrophy. If the nerve injury is distal to the sensory supraspinatus nerve fibers, there will not be a history of shoulder pain [41]. On the other hand, supraspinatus and infraspinatus atrophy suggest a more proximal compression [10]. Supraspinatus fossa atrophy is considered a pathognomonic sign of suprascapular entrapment [4,11]. Additionally, tenderness over the suprascapular or spinoglenoid notch may be found [34]. Clinical evidence shows that adduction and internal rotation can exacerbate pain because this position stretches the spinoglenoid ligament, increasing nerve compression [9]. Lafosse has described the suprascapular stretch test. While standing behind the patient, the clinician gently turns the patient’s head to the side opposite to the shoulder that needs to be evaluated while pressing down on the patient’s shoulder with the other hand. This test is positive if the patient reports pain at the posterior aspect of the shoulder (Figure 3A) [23,29]. Another helpful test is the “cross arm adduction test” described by Plancher. The patients are asked to over adduct and internally rotate the arm. If the patient refers to an exacerbation of the pain at the posterior aspect of the shoulder, the test is positive (Figure 3B). Nerve compression at the suprascapular notch may lead to over a 75% loss in strength in abduction and external rotation [10]. Conversely, entrapment at the spinoglenoid notch is better tolerated thanks to the compensation mechanisms of the muscles in the shoulder region involving the deltoid and infraspinatus muscles. A helpful way to differentiate the suprascapular entrapment from other shoulder pathologies is the local injection of anesthetic in the notch. The use of fast-acting anesthetic is strongly recommended. It may be injected into the spinoglenoid notch to confirm the diagnosis of suprascapular nerve entrapment. The needle is placed 4cm medial to the posterolateral corner of the acromion. Plancher et al. suggested performing US ultra-sound guided injection into the spinoglenoid notch to increase precision. This test is positive if the patients reports pain relief. Additionally, a cross-arm adduction test is then performed with reduced or no pain.

### 2.4. Radiological Evaluation

**Figure 4:**
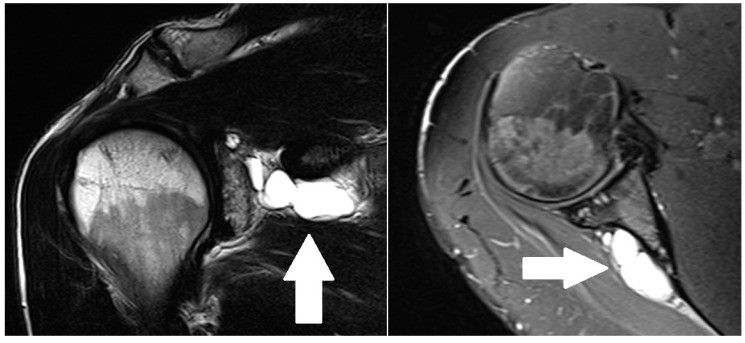
Patient affected by paralabral cyst of the right shoulder: coronal (left image) and transverse (right image) views of an MRI with contrast agent. The arrows highlight the cyst.

The medical history and clinical evaluation must guide any request for diagnostic studies. If suprascapular neuropathy is suspected, plain radiographs are mandatory. Standard X-rays like the anteroposterior, lateral, axial, Grashey and scapular outlet views may reveal concomitant shoulder diseases. The “Stryker notch” view is a specific view for suprascapular and spinoglenoid notch evaluation that should be included in the radiographic evaluation (Table 2). Additionally, the Zanca view can be useful to evaluate the acromioclavicular joint [39]. A CT scan may be useful to detect bone lesions like fractures or anatomic variations that might compress the nerve. An MRI is the best way to identify space occupying lesions (Figure 4), to assess supra-spinatus and infra-spinatus muscles atrophy and to examine the course of the nerve through bony prominences [10,28]. Three main signs must be looked for in the MRI. These are edema, atrophy and fatty degeneration of the muscles. Edema of the supra-spinate and infra-spinate muscles is a pathognomonic sign of suprascapular neuropathy. It may be detected some days after a trauma or, in some cases, at the onset of electromyography (EMG) abnormalities. Generally, muscle edema continues to increase after a trauma, reaching a maximum after 2–4 weeks [28,41]. Its intensity and duration are proportional to the severity and the onset of the lesion. However, neuropathy-related muscle edema in sportspeople can be seen even more than 6 months after the trauma. While muscle atrophy usually occurs quickly, fatty degeneration of the muscles has a late onset. Both are less useful signs in the evaluation of the neuropathy because they are not predictive of the severity [28]. Mucoid cysts are the most frequent space occupying lesions causing secondary compression of the suprascapular nerve. They are linked to three main shoulder lesions, the type 2 SLAP lesion, the postero-superior impingement syndrome or posterior instability. Mucoid cysts are usually clearly identified with ultrasounds, but these lesions always communicate with the joint. Therefore, they can be easily found by means of MRI-arthrography or CT-arthrography. Unfortunately, some lesions may be undetectable because of obstructed communication with the joint and could lead to a missed diagnosis. Finally, CT angiography can be useful in some rare cases caused by suprascapular artery compression due to its anatomical variation [28]. A further method for studying the morphology and the course of the nerve is the high-resolution 3T MR Neurography. This technique is quite helpful. Sadly, it is not widely available. It can directly show the nerve abnormality as well as secondary muscle denervation changes. [42].

### 2.5. Electrodiagnostic Tests

The gold standard diagnostic tests for suprascapular neuropathy that allows for the detection of the level of the injury are the EMG and nerve conduction velocity (NCS) tests [10]. They have from 70% to more than 90% sensitivity in detecting muscle fiber denervation [10,23,32,34]. The main findings in peripheral neuropathies are an increase in motor pulse latency and signs of denervation like fibrillation and sharp waves. Occasionally, chronic neuropathic shoulder pain has negative results in electrodiagnostic tests [23]. It may be because they do not detect “small fiber” damage (i.e., unmyelinated and thinly myelinated pain fibers) [4,11].

### 2.6. Treatment

The etiology of suprascapular neuropathy is the most relevant factor in choosing the appropriate treatment [10]. There is no consensus on the first steps in the treatment of suprascapular nerve entrapment. However, most authors advise starting suprascapular neuropathy treatment non-operatively unless nerve compression is caused by a mass [10,32]. If non-operative treatment fails, surgical decompression is recommended. Furthermore, Plancher et al. suggest non-operative treatment for a minimum time for patients who present a visible atrophy of the infra-spinatus. Regardless, the optimal duration of non-operative treatment remains unclear [32,39]. When there is no atrophy, no remarkable finding on an electromyogram (EMG), and no evidence of a labral tear or ganglion cyst, but weakness and pain are present, the recommendation is a 6-month course of non-operative treatment before considering any type of operative intervention [32].

#### 2.6.1. Non-Operative Treatment

It requires avoiding overhead activity, but a shoulder rehabilitation program and anti-inflammatory therapy with NSAIDs should be followed. Physical exercise must focus on shoulder flexibility and on periscapular and deltoid musculature strengthening. Rehabilitation exercises must be closely monitored in order to avoid any further injury to the nerve during its healing process. Evidence shows that non-operative treatment tends to have bad outcomes in patients whose symptoms continue for more than 6 months and in patients with muscle atrophy, space occupying lesions and massive rotator cuff tears. In such cases, early surgical intervention may be considered because the delay in nerve decompression could result in incomplete restoration of muscle function. The reasons why patients improve with non-operative treatment is not known but it may be explained by compensatory muscle mechanisms [10,41]. The success rate of non-operative treatment remains unclear.

#### 2.6.2. Operative Treatment

Indications for surgical treatment are nerve entrapment by space occupying lesions and the failure of nonoperative management [10]. It has been demonstrated that patients with soft tissue suprascapular nerve entrapment experience better outcomes with surgical treatment and arthroscopic intervention should be preferred to open techniques, when possible [10,23,32,43,44]. Cyst ganglion aspiration under ultrasound or CT guidance carries a 75% to 100% rate of failure or recurrence. Therefore, surgery is indicated [10,32]. There is no consensus as to whether a labral lesion and ganglion cyst should be treated all together or not [32]. Some authors recommend labral repair and cyst decompression in the same procedure [45,46]. However other authors have argued that labral repair without cyst decompression was the most appropriate treatment [47,48]. Some authors recommend wide arthroscopic decompression of the suprascapular nerve at both the spinoglenoid and the suprascapular notches in patients with dynamic nerve compression [8,10,49]. Finally, Lafosse recommends surgical release of the suprascapular nerve in symptomatic patients regardless of the electromyography findings given that it is a quick and safe procedure with great potential benefits for the patients. He underlined that suprascapular nerve entrapment is often a dynamic condition, so it is not always seen in an electromyography [23]. 

#### 2.6.3. Open Approach

Open suprascapular notch decompression: a transverse skin incision parallel with the edge of the scapular spine or a curved incision medial to the acromioclavicular joint is made. The trapezius muscle is split by blunt dissection. The supraspinatus muscle is then identified and dislocated posteriorly. The suprascapular notch is then identified at the base of the coracoid. The suprascapular neurovascular bundle and the transverse scapular ligament can be seen. The localization of the neurovascular bundle must be performed with care because of its anatomical variations. The transverse scapular ligament is then released, and the nerve can be freed. If necessary, further nerve decompression can be obtained by a deepening or widening of the notch [10,35,50,51].

Open spinoglenoid notch decompression: a skin incision is made starting approximately 3 cm medial to the posterolateral corner of the acromion and then extended downward toward the posterior axillary skin fold. The deltoid muscle is split by blunt dissection. The fascia is opened and the infraspinatus muscle belly is dislocated inferiorly to expose the spinoglenoid notch. A consistent ligamentous structure from the lateral side of the scapular spine to the posterior aspect of the shoulder capsule is now visible. This spinoglenoid ligament is cut from the edge of the scapular spine. The nerve can be released by using scissors proximally and blunt dissection inferiorly, taking care to avoid nerve and vessel injuries. If a ganglion cyst is present, it is well exposed after ligament release and can be excised [44].

#### 2.6.4. Arthroscopic Approach

Although the arthroscopic suprascapular nerve release technique is a relatively novel technique, it must be considered the gold standard [5,10,23,29,33,43,52]. Most of the work on this procedure has been carried out on cadaveric specimens but many authors have demonstrated the procedure’s safety in experienced hands [10]. Hemostasis is critical during the whole procedure. Adequate pump pressure (50–55 mm Hg) and hypotensive anesthesia (systolic blood pressure, 100–110 mm Hg) are recommended [43].

**Figure 5:**
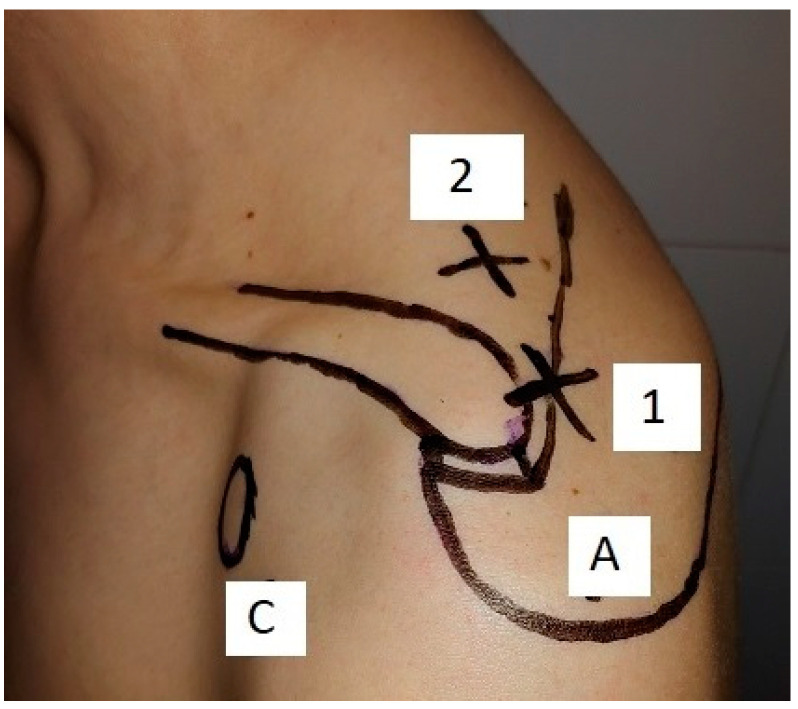
Schematic top view of the right shoulder with the refferal point for establishing Neviaser (blue cross) and LaFosse (green cross) portals. C: coracoid, SN: suprascapular nerve, SA: suprascapular artery, SNo: suprascapular notch, SGNo: spino-glenoid notch.

**Figure 6:**
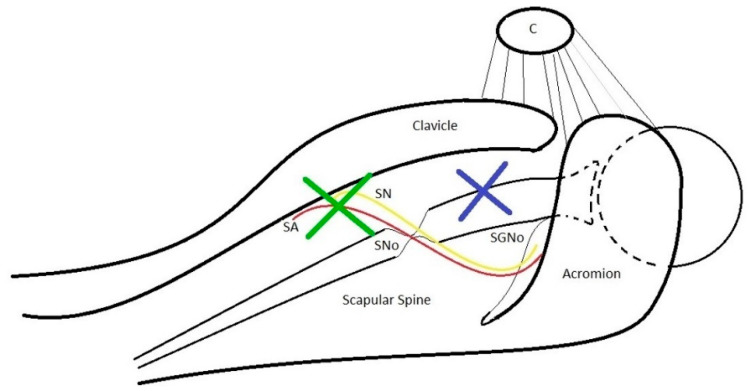
Neviaser portal (1) is placed approximately 1 cm medial to the acromion, between the clavicle and the scapular spine. LaFosse portal is located 2-3 cm medial to the Neviaser portal. Coracoid (C), acromion (A).

Suprascapular notch compression: with the patient in the beach chair position, a standard diagnostic arthroscopy is performed using a posterior portal. Plancher et al. suggest avoiding doing glenohumeral joint inspection first or carrying it out as briefly as possible to prevent joint swelling [32,39]. An arthroscopic trocar is then gently introduced through the suprascapular nerve portal of Lafosse. While creating this portal, attention must be paid to the suprascapular nerve and artery, which are located about 2 mm medial to this portal (Figure 5 and Figure 6). A blunt dissection is then carried out around the transverse scapular ligament, taking care to avoid artery and nerve damage. Then, arthroscopic scissors are introduced through the Neviaser portal from where the transverse scapular ligament can be cut, thus releasing the suprascapular nerve. As described by Krishnan et al., the suprascapular nerve can be visualized and decompressed anteriorly to the coracoclavicular ligaments [11,23,43,53]. If further decompression is needed, deepening and widening of the suprascapular notch (notchplasty) can be performed. Some authors advise against notchplasty with a burr because of the possibility of excessive postoperative scar tissue and recurrent compression of the nerve [23]. After nerve release, other concomitant lesions can be treated [10,29,52]. As previously stated, the arthroscopic procedure is now considered the gold standard technique. It is a quick and safe procedure in experienced hands. The learning curve is not predictable as it is a not frequent performed surgery. Very good clinical results have been described, especially in those patients with no signs of atrophy. Most of the patients can return to sport activities at the same level or higher within 8 months [8,10,22,39].

Spinoglenoid notch decompression: the patient is prepared in the beach-chair position. Two portals must be made: the viewing portal and the working portal. They are placed respectively 8 cm and 4 cm medially to the posterolateral corner of the acromion, just inferior to the scapula spine. The trocar is introduced into the viewing portal and directed towards the infraspinatus fossa. The trocar is then directed close to the working portal into the spinoglenoid notch. At this point, to ensure optimal visualization, Plancher et al. suggest sweeping the trocar under the roof of the infraspinatus spine feeling the curvature. Then, the trocar is introduced into the working portal in order to clear the soft tissue and the spinoglenoid ligament using a radiofrequency tool or non-aggressive shaver with the suction turned off. The ligament can be resected and the nerve safely released [32].

Complications after arthroscopic suprascapular nerve release are uncommon. Special care must be taken during dissection, shaving or electrocautery to avoid iatrogenic injury to the suprascapular nerve and to the relative vessels. Anatomic variations may be present and must be recognized [11,43]. Postoperative rehabilitation is guided by concomitant pathology. All patients must utilize a sling postoperatively for comfort. In cases of dynamic suprascapular neuropathy at the spinoglenoid notch, patients can start passive shoulder exercise and Codman’s exercise immediately [44]. In patients with a surgical labral repair, wearing a sling for 3 weeks is advisable. Shoulder strengthening exercises can start at 4–8 weeks postoperatively in cases of isolated suprascapular nerve decompression at the spinoglenoid notch or when shoulder motion is at least 80% of the contralateral shoulder in the other cases [11].

## 3. Results

In the cases of dynamic primary compression, the overall response to conservative management has good outcomes. Pain relief is achievable in more than 80% of the cases and almost all the patients can return to sport activities within 3 months [10]. After evaluating all clinical features, including the course of follow-up, the timing of return-to-sport activity must be carefully planned, considering the differences in patient characteristics. In patients with painless infraspinatus muscle atrophy without a space occupying lesion, function is usually recovered with non-operative care. While significant pain relief is achieved in 80% to 96% of patients, regaining muscle strength and function and atrophy is less predictable [10,54]. After surgical suprascapular notch decompression, most reports show a good return in terms of pain relief and an improvement in supraspinatus muscle strength. Some authors have found that 80% of the treated patients showed improvement in the strength and atrophy of the supraspinatus muscle, but only 50% showed improvement in the infraspinatus [9,10,39]. In the cases of spinoglenoid notch nerve compression, several authors have reported excellent results after operative management, as well. Authors described their own experience, in which the most satisfied patients (97%) were treated with labral repair and open or arthroscopic decompression of the cyst, whereas the less satisfied patients were those treated with isolated labral repair (67%) or needle aspiration of the cyst (64%), or in people treated non-operatively (53%) [2,9]. The outcomes of nerve release and rotator cuff repair vary widely as the degree of fatty degeneration of the rotator cuff, the reciprocal influence of suprascapular nerve palsy and rotator cuff tendon tears on have muscle pathology are unclear. Debate continues as to how retracted cuff tears can lead to suprascapular neuropathy and whether suprascapular palsy or tendon retraction cause muscle fat degeneration [8,13,26,55]. In all the cases without a clear mechanical cause for suprascapular neuropathy, conservative treatment can usually best resolve the symptoms (Table 3) [56,57,58,59].

## 4. Conclusions

Suprascapular nerve entrapment is an increasingly recognized and diagnosed shoulder lesion, but it is still rather infrequent. Diagnosis may be complex, whereas its treatment is safe, and it has a great success rate. Prompt diagnosis is of utmost importance as chronic conditions have worse outcomes compared to acute lesions. The medical history and clinical evaluation conducted with proper instrumental evaluation and imaging are essential. Dynamic compression must initially be treated non-operatively. If there is no improvement, surgical release should be considered. On the other hand, soft tissue lesions may first be treated non-operatively. However, surgical treatment by arthroscopic means is advisable when possible as it represents the gold standard therapy. Other concomitant shoulder lesions must be recognized and treated accordingly.

## Figures and Tables

**Table 1:** Summary of the main secondary etiologies.

Secondary Aetiologies	Examples
Space occupying lesions	Neoplasm, ganglion cyst, ossified scapular ligament
Traumatic conditions	Scapula fractures, shoulder dislocation, massive cuff tear, distractive trauma, penetrating trauma
Post traumatic disorders	Hematomas, heterotopic ossification, scars
Systemic conditions	Pregnancy, diabetes mellitus, hypertiroidism
Iatrogenic conditions	Arthroscopic tear cuff repair, Latarjet procedure

**Table 2:** Name, description and sample of the main specific x-ray views.

X-ray Views	Description	Sample
**Grashey view** (or AP oblique shoulder view)	Obtained with the patient rotated 35–45° towards the affected shoulder or the beam has to be tilted laterally from the standard AP view.	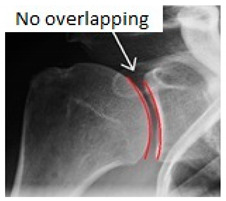
**Stryker notch view**	The patient is in supine position; the upper limb is abducted and externally rotated with the hand supporting the back of the head and the elbow pointed towards the ceiling. X-ray beam is angled 10° cephalad, centered on the coracoid process.	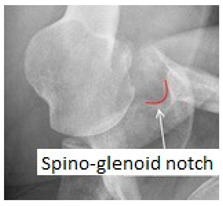
**Scapular outlet view**	The patient is placed in 60° anterior oblique position. The arm is left in neutral position or in the sling. By feeling the scapula, adjust the position to get the scapula perpendicular to the film.	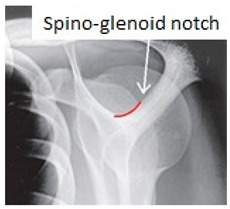

**Table 3:** Main diagnosis with suggested treatment.

Condition	Treatment
Pain and weakness with no atrophy, no remarkable findings on electromyogram (EMG), and no relevance of labral tear or ganglion cyst	6months conservative treatment. If it fails, consider surgical arthroscopic nerve release
Patients with visible atrophy on physical examination	Attemption of conservative treatment for a minimum of 1 month; the optimal length of non-operative treatment remains unclear (32,39). After that, consider the surgical treatment
Space occupying lesion compression	Surgical treatment. In case of ganglion cyst diagnosis, best result are given by labral repair and open or arthroscopic decompression of the cyst (2,9)
